# Nonadiabatic superconductivity in a Li-intercalated hexagonal boron nitride bilayer

**DOI:** 10.3762/bjnano.11.102

**Published:** 2020-08-07

**Authors:** Kamila A Szewczyk, Izabela A Domagalska, Artur P Durajski, Radosław Szczęśniak

**Affiliations:** 1 Division of Theoretical Physics, Jan Długosz University in Częstochowa, Ave. Armii Krajowej 13/15, 42-200 Częstochowa, Poland; 2 Quantum Optics and Engineering Division, Faculty of Physics and Astronomy, University of Zielona Góra, Prof. Z. Szafrana 4a, 65-516 Zielona Góra, Poland; 3 Division of Physics, Częstochowa University of Technology, Ave. Armii Krajowej 19, 42-200 Częstochowa, Poland

**Keywords:** critical temperature, electron–phonon interaction, Li-hBN bilayer, Li-intercalated hexagonal boron nitride (Li-hBN), nonadiabatic superconductivity, vertex corrections

## Abstract

When considering a Li-intercalated hexagonal boron nitride bilayer (Li-hBN), the vertex corrections of electron–phonon interaction cannot be omitted. This is evidenced by the very high value of the ratio λω_D_/ε_F_ ≈ 0.46, where λ is the electron–phonon coupling constant, ω_D_ is the Debye frequency, and ε_F_ represents the Fermi energy. Due to nonadiabatic effects, the phonon–induced superconducting state in Li-hBN is characterized by much lower values of the critical temperature (*T*^LOVC^_C_ ∈ {19.1, 15.5, 11.8} K, for μ* ∈ {0.1, 0.14, 0.2}, respectively) than would result from calculations not taking this effect into account (*T*^ME^_C_∈ {31.9, 26.9, 21} K). From the technological point of view, the low value of *T*_C_ limits the possible applications of Li-hBN. The calculations were carried out under the classic Migdal–Eliashberg formalism (ME) and the Eliashberg theory with lowest-order vertex corrections (LOVC). We show that the vertex corrections of higher order (λ^3^) lower the value of *T*^LOVC^_C_ by a few percent.

## Introduction

Low-dimensional systems such as graphene [1–5], silicene [6], borophene [7–8], and phosphorene [9–11] are mechanically stable only when placed on a substrate [12–14]. The substrate should be selected so that it changes the physical properties of the low-dimensional system as little as possible. In the case of graphene, the following substrate materials were used: Co [15], Ni [16–19], Ru [20–21], Pt [22–23], SiC [24–26], and SiO_2_ [27–29]. Unfortunately, the obtained experimental data showed that the incompatible crystalline structure of the above materials leads to significant suppression of the carrier mobility of graphene [13,30].

It is now assumed that the best substrate for graphene is hexagonal boron nitride (hBN) with a honeycomb crystal structure in which boron (B) and nitrogen (N) atoms alternatingly occupy the hexagonal lattice nodes. In the bulk form, hBN was synthesized by Nagashima et al. in 1995 [31]. A decade later, the two-dimensional form of hBN was obtained at the University of Manchester [32].

Monolayers of graphene and hBN have a very similar crystal lattice structure. Their compatibility is estimated to be 98.5% [23]. In a graphene/hBN composite, a homogeneous distribution of charge on the graphene surface is observed. This result is radically different from the data obtained for graphene/SiO_2_ [33]. In addition, hBN monolayers exhibit a high temperature stability, a low dielectric constant (ε = 3–4), and a high thermal conductivity [34]. The band gap of hBN is about 5.9 eV [35]. Furthermore, which is also important, hBN is nontoxic.

It is worth noting that graphene on a hBN substrate was used to fabricate transistor devices with high mobility [35], with the help of which the quantum Hall effect was observed. A heterojunction with two graphene layers [30] and superlattice structures [36–38] were also constructed. The graphene/hBN heterojunction devices allowed for the detection of the Hofstadter’s butterfly phenomenon [39–40]. In both layer and bulk form, hBN has a large bandgap energy, which makes it an insulator [13,41]. Therefore, for a long time this material was not associated with superconductivity. The situation changed when it was suggested that the intercalation of lithium in hBN induces a transition to the metallic state [42]. Quasi-two-dimensional superconducting systems are currently being intensively studied for possible applications in nanometerscale superconducting quantum interference devices [43] and quantum information technology [44–45].

Currently, the most promising research seems to be the properties of the superconducting state in Li-intercalated hexagonal boron nitride bilayer (Li-hBN) compounds. Based on DFT calculations, it has been shown that the critical temperature (*T*_C_) of the superconductor–metal phase transition is about 25 K [41] for the Coulomb pseudopotential μ* = 0.14 (identical to the experimental value of μ* obtained for graphene [46]). The expected value of *T*_C_ is much higher than the maximum temperature that was achieved in graphene intercalated with alkali metals (*T*_C_ = 8.1 K in Ca-intercalated bilayer graphene) [5]. Also, this value is higher than that of other superconducting low-dimensional structures, e.g., *T*_C_ ≈ 20 K for a Li- and Na-intercalated blue phosphorene bilayer [47], *T*_C_ ≈ 16.5 K for a Li-intercalated black phosphorene bilayer [48], and *T*_C_≈ 10 K for a Li–MoS_2_ bilayer [49]. The obtained result for Li-hBN is explained by the relatively high value of the electronic density of states at the Fermi level and the significant contribution to the pairing interaction from the inter-layer electron–phonon coupling [41]. This is due to the formation of characteristic bonds connecting two boron atoms in the upper and lower layers of hBN, which results from the low electronegativity of boron atoms.

From the experimental point of view, it is worth paying attention to the results of research conducted in 2019 by S. Moriyama and co-workers [50]. A superconducting state has been observed in a system consisting of non-twisted bilayer graphene (BLG) and hexagonal boron nitride layers (hBN/BLG/hBN). The following characteristic temperatures were obtained: *T*^onset^ ≈ 50 K, T* ≈ 30 K, and *T*_BKT_ = 14 K, which correspond the onset of superconductivity (90% of the total transition/normal resistance), the crossover to superconductivity (50% of the normal resistance), and the confinement of vortices, respectively.

The important question is whether the Li-hBN bilayer system yield the high critical temperature that was suggested from DFT calculations (*T*_C_ = 25 K) [41]. We think that this not the case because electron–phonon interaction in Li-hBN needs to be taken into account together with vertex corrections. This is demonstrated by the very high ratio of λω_D_/ε_F_ ≈ 0.46, where λ = 1.17 is the electron–phonon coupling constant, ω_D_ = 165.56 meV is the Debye frequency, and ε_F_ = 417.58 meV represents the Fermi energy [41]. Thus, in the presented paper, we characterized the properties of the superconducting state in a Li-hBN bilayer in the framework of the Eliashberg formalism, which includes the vertex corrections of electron–phonon interaction [51]. We compared the results with those obtained using the classical Migdal–Eliashberg theory [52]. Note that the use of the Eliashberg formalism is associated with the high value of the electron–phonon coupling constant λ, which characterizes the superconducting state in Li-hBN [41]. Let us remind that the BCS theory gives the correct results only in the weak-coupling limit, where λ *<* 0.3 [53–54]. The scope of applicability of the Migdal–Eliashberg theory is carefully discussed in [55].

## Theoretical Model

The classical Migdal–Eliashberg (ME) formalism [52,56] represents the natural generalization of the BCS theory (the first microscopic theory of the superconducting state) [53–54]. This generalization takes into account the retardation and strong-coupling effects of the electron–phonon interaction, which are responsible for the condensation of electrons in Cooper pairs [57]. As part of the Eliashberg formalism, the electron–phonon interaction is quantified by the so-called Eliashberg function (α^2^*F*(ω)). The form of the Eliashberg function for a specific physical system can be determined theoretically through DFT calculations [58], or experimentally using the data provided by tunnel experiments [59–60]. The electron correlations (the screened Coulomb interaction) are modeled parametrically defining the so-called Coulomb pseudopotential (μ*) [61]. The Eliashberg function and μ* are the only input parameters of the isotropic Eliashberg equations.

The classical Eliashberg equations are thoroughly discussed in the literature [62]. They allow for the self-consistent determination of the superconducting order parameter (Δ*_n_* = Δ(iω*_n_*) and the wave function renormalization factor (*Z_n_* = *Z*(iω*_n_*), with an accuracy of the second order relative to the electron–phonon coupling function (*g*). The symbol ω*_n_* = π*k*_B_*T*(2*n* + 1) defines the fermionic Matsubara frequency. In the case of the phonon-induced superconducting state, the limitation of considerations to the order of *g*^2^ is justified by the Migdal theorem [56]. The Migdal theorem applies when the ratio λω_D_/ε_F_ is of the order of 0.01. This means that the energy of the phonons is so small that the vertex corrections for the electron–phonon interaction are irrelevant.

According to DFT calculations, the value of the ratio λω_D_/ε_F_ for Li-hBN is 0.46. This is why the superconducting state in Li-hBN cannot be quantified in the classical Eliashberg theory. The unusually high value of the λω_D_/ε*_F_* ratio for Li-hBN is related to the fact that the physical system is quasi-two-dimensional. In the case of the bulk superconductor, the width of the electron band is significantly broadened, which results in the increase of the Fermi energy (ε_F_ = 1.63 eV). In addition, the electron–phonon coupling constant decreases (λ = 0.66). As a result, λω_D_/ε_F_ is only 0.07. The calculations carried out by us within the Migdal–Eliashberg formalism prove that the superconducting state has a significantly lower critical temperature value in the bulk than in the quasi-two-dimensional system. In particular, we obtained *T*_C_ ∈ {14.01, 8.64, 4.6} K, for μ* ∈ {0.1, 0.2, 0.3}, respectively.

To realize how uncommonly high the value of λω_D_/ε_F_ for Li-hBN is, it is enough to note that for the Li–MoS_2_ bilayer, we obtain λω_D_/ε_F_ = 0.15 [49]. In bilayers of black and blue phosphorus intercalated with lithium, λω_D_/ε_F_ is equal to 0.05 and 0.1, respectively [47–48]. A value of λω_D_/ε_F_ of 0.09 causes a noticeable modification of the properties of the superconducting state, as in the case of LiC_6_, where *T*_C_ ≈ 6 K [2,46,63–64].

Therefore, to describe the superconducting state in Li-hBN, we used the Eliashberg equations derived with an accuracy of the fourth order relative to *g* (lowest-order vertex corrections, LOVC). These equations were derived by Freericks et al. [51] to analyze the properties of the superconducting state in lead. They take the form given in Equation 1 and Equation 2 (*A* = 1):

[1]
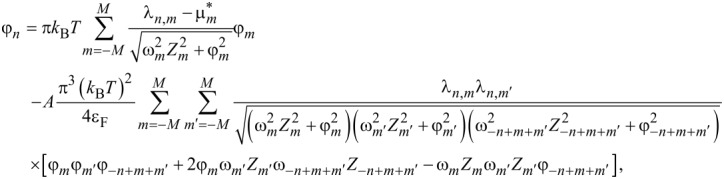


[2]
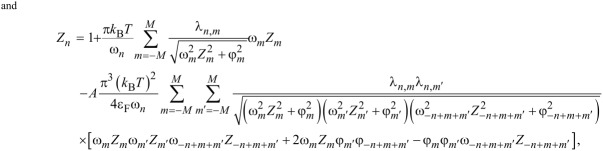


while for *A* = 0, we get the classic Migdal–Eliashberg equations. The order parameter is given by the formula Δ*_n_* = φ*_n_*/*Z_n_*. The symbol λ*_n,m_* represents the pairing kernel for the electron–phonon interactions:

[3]
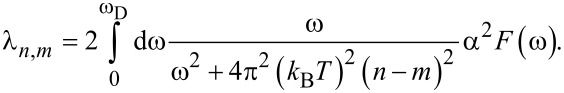


The Coulomb pseudopotential function is: 
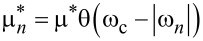
, where θ(*x*) is the Heaviside function, and ω_c_ represents the cut-off frequency (ω_c_ = 3ω_D_ = 496.7 meV).

Freericks’ equations allow one to determine the values of the order parameter and the wave function renormalization factor in a self-consistent manner, which is undoubtedly their great advantage. These are isotropic equations, which means that the self-consistent procedure does not apply to the electron momentum (**k**). From the physical point of view this should not be significant, because the phonon-induced superconducting state is highly isotropic [62]. The situation would of course change radically if, in addition, the strong electron correlations had to be taken into account. Eliashberg equations including vertex corrections and an explicit dependence on **k** are also given in the literature [65–67]. These equations were derived in the context of research on the superconducting state in fullerene systems [68–69], in high-*T*_C_ cuprates [70–72], in heavy fermion compounds [73], and in superconductors under high magnetic fields [74]. Unfortunately, due to enormous mathematical difficulties, their full self-consistent solutions are still unknown (Δ*_n_*_,_**_k_** and Z*_n_*_,_**_k_**).

Also, Freericks’ equations have been recently successfully used to analyze the superconducting state with high critical temperature values in compounds such as PH_3_ (*T*_C_ ≈ 80 K), H_3_S (*T*_C_ ≈ 200 K) [75], and H_2_S (*T*_C_ ≈ 35 K) [76].

From the mathematical point of view, the Eliashberg equations are solved in a self-consistent manner taking into account the correspondingly large number of fermionic Matsubara frequencies [77–78]. In our considerations, we assumed that this number (*M*) is 4000, which ensured the appropriate convergence of solutions of the Eliashberg equations for a temperature higher or equal to *T*_0_ = 4 K. Due to the lack of experimental data in the examined physical system we took into account the Coulomb pseudopotential in a range from 0.1 to 0.2, while the value of 0.14 was already considered in [41].

## Results

**Figure 1:**
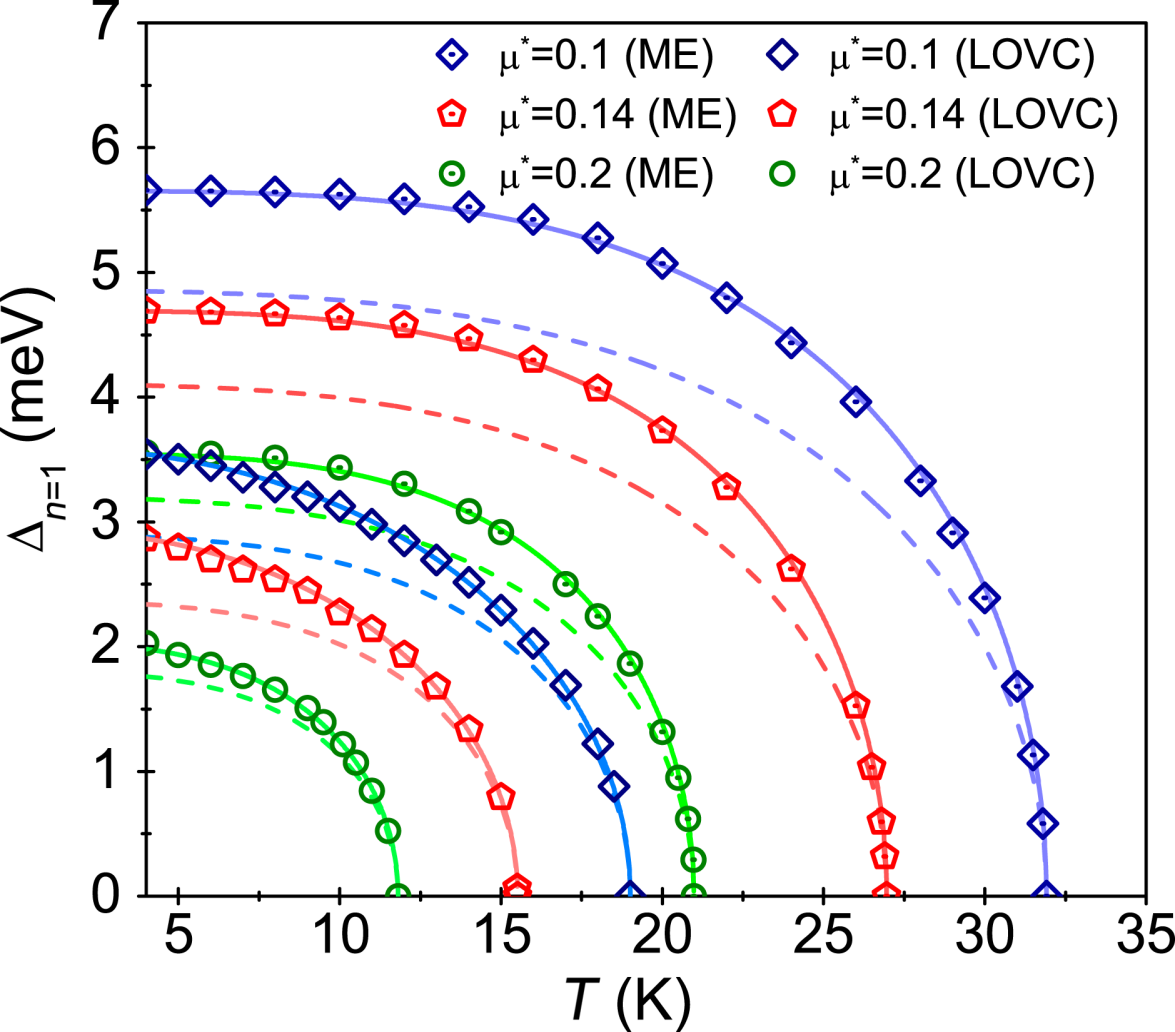
The order parameter as a function of the temperature. ME model - symbols with the dot, LOVC model - empty symbols, μ* ∈ {0.1, 0.14, 0.2}. The solid lines represent the parameterization of numerical results using Equation 4. The dashed lines were obtained as part of the BCS theory (mean-field theory).

In Figure 1, we plotted the dependence of the order parameter on the temperature. Note that under the imaginary axis formalism, it is assumed that the physical value of the order parameter is Δ*_n_*_=1_. In the classic ME model, we obtained the following critical temperature values: 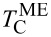 ∈ {31.9, 26.9, 21} K, respectively, for μ* ∈ {0.1, 0.14, 0.2}. Comparing the obtained results with the results taking into account the impact of the vertex corrections, 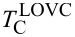 ∈ {19.1,15.5,11.8} K), we find that the nonadiabatic superconducting state in Li-hBN has a much lower value of *T*_C_ than it would follow from the ME model.

The observed lowering of the critical temperature value does not only result from the static corrections (Stat.), a good measure of which is the ratio *m* = ω_D_/ε_F_ = 0.4 (Migdal parameter). It is also associated with dynamic corrections modeled by the explicit dependence of the order parameter and the wave function renormalization factor on the Matsubara frequency. Based on the results of [67,79], the impact of static vertex corrections on the critical temperature can be estimated using the formula

[5]
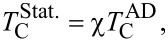


where 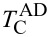 is the critical temperature value calculated on the basis of the Allen–Dynes formula [80]. The input from the static part of the vertex corrections has the form

[6]
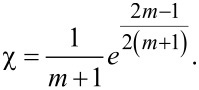


A good measure of the dynamic vertex corrections is


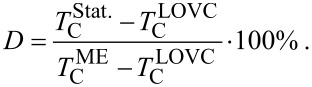


The results are summarized in Table 1. As one can see, the static part of the vertex corrections is responsible for 80–90% of the difference in *T*_C_.

**Table 1:** Critical temperature estimated from the LOVC model, from the ME model, using the Allen–Dynes formula [80], and from the analytical model including static corrections (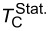). Additionally, the values of the *D* parameter were given.

μ*	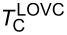 (K)	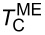 (K)	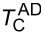 (K)	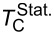 (K)	*D*%

0.1	**19.1**	31.9	32.2	**21.4**	**18**
0.14	**15.5**	26.9	26.7	**17.8**	**20.2**
0.2	**11.8**	21	19.4	**12.9**	**12**
					

The numerical results obtained from the Eliashberg equations can be parameterized using the formula [81]

[4]
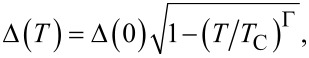


where Δ(0) = Δ(*T*_0_). Using the LOVC model, we obtained Γ ∈ {2.17, 2.2, 2.8}, respectively, for μ* ∈ {0.1, 0.14, 0.2}. The exponent Γ for the classic ME approach differs significantly in values, i.e., Γ∈ {3.45, 3.4, 3.45}, respectively. The accuracy of analytical parameterization of the numerical results is presented in Figure 1 (solid lines). In addition, the results obtained under the mean-field BCS model are given by the dashed lines. In this case, Δ(0) = 1.76·*k*_B_*T*_C_ was adopted [53–54]. The value of the exponent Γ for the BCS model is 3 [81].

Note the differences in the shape of the curves corresponding to the parameterization of the Eliashberg results and the BCS theory. In the case of the ME model, the differences result only from retardation and strong-coupling effects correctly taken into account in the ME formalism. These effects can be characterized by calculating the value of the ratio *r* = *k*_B_*T*_C_/ω_ln_, where


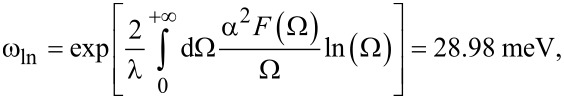


is the logarithmic phonon frequency [80]. The *r* parameter for Li-hBN is *r*^ME^∈ {0.095, 0.08, 0.062 } or *r*^LOVC^∈ {0.057, 0.046, 0.035}, respectively, for μ* ∈ {0.1, 0.14, 0.2}. This means that the effects considered are significant even when we consider the vertex corrections for the electron–phonon interaction. Also note that retardation and strong-coupling effects for Li-hBN are of the same order as in Li-MoS_2_ bilayer compounds [49], Li-black phosphorene bilayers [48], and Li-blue phosphorene bilayers [47], i.e., 0.068, 0.094, and 0.099, respectively. These results were obtained for *T*_C_ determined from the Allen–Dynes formula [80] assuming μ* = 0.1. In the BCS limit, the Eliashberg equations predict *r*→0.

**Figure 2:**
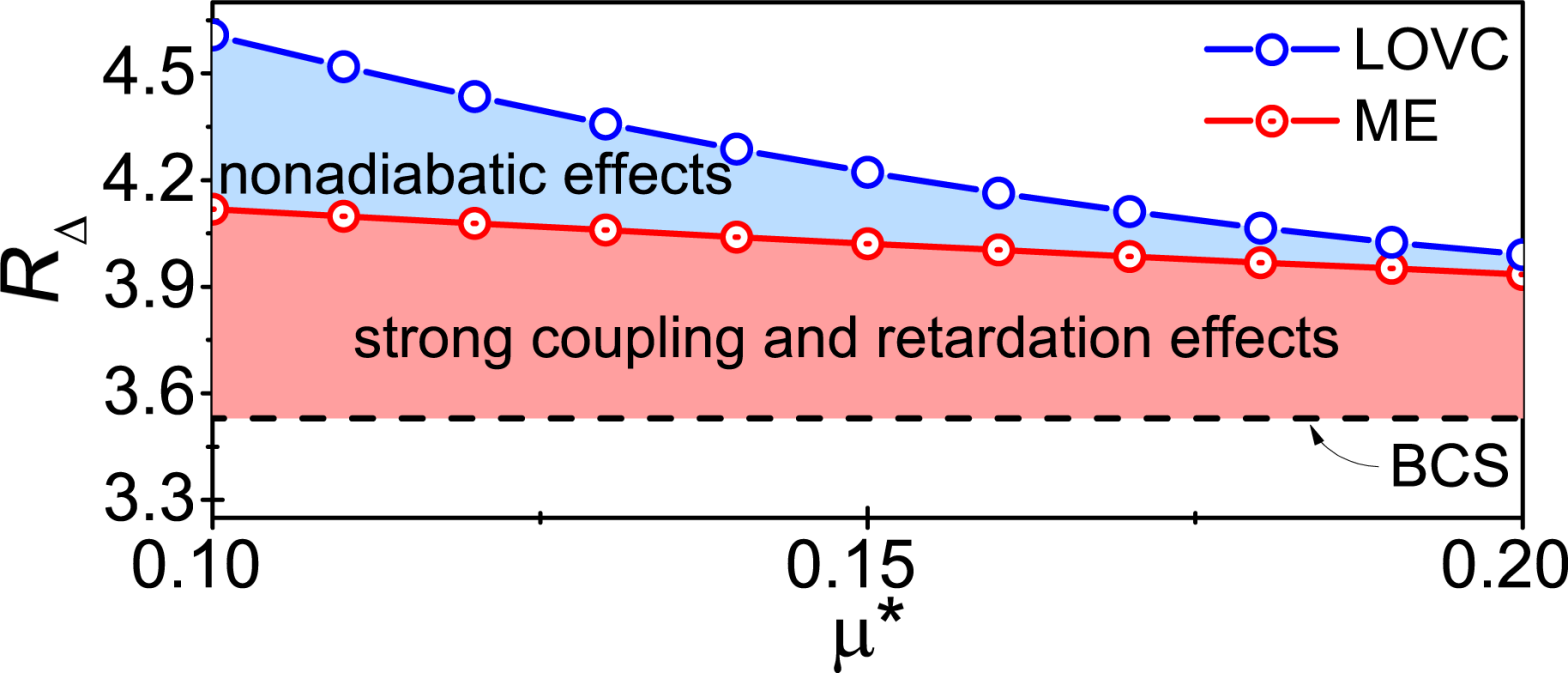
The values of the ratio *R*_Δ_ as a function of the Coulomb pseudopotential. The results obtained under the model: LOVC, ME, and BCS.

In the LOVC theory, we take into account the vertex corrections as well as the retardation and strong-coupling effects. As a result, the differences between the Eliashberg parameterization curves and the BCS curves noticeably increase. A good measure of this effect is the value of the ratio *R*_Δ_ = 2Δ(0)/*k*_B_*T*_C_. For the Li-hBN system, we obtained 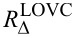 ∈ {4.6, 4.29, 3.99} and 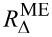 ∈ {4.12, 4.04, 3.9}. It should be emphasized that using BCS theory, a value of *R*_Δ_ = 3.53 is obtained. It is the universal constant of the model [53–54]. The results obtained for μ* ∈ ⟨0.1, 0.2⟩ are presented in Figure 2. One can notice an interesting effect, namely, that with the increase of depairing electron correlations, the impact of vertex corrections on the ratio *R*_Δ_ decreases, i.e., for μ* ≈ 0.2 the parameter 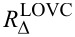 differs only slightly from 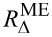.

**Figure 3:**
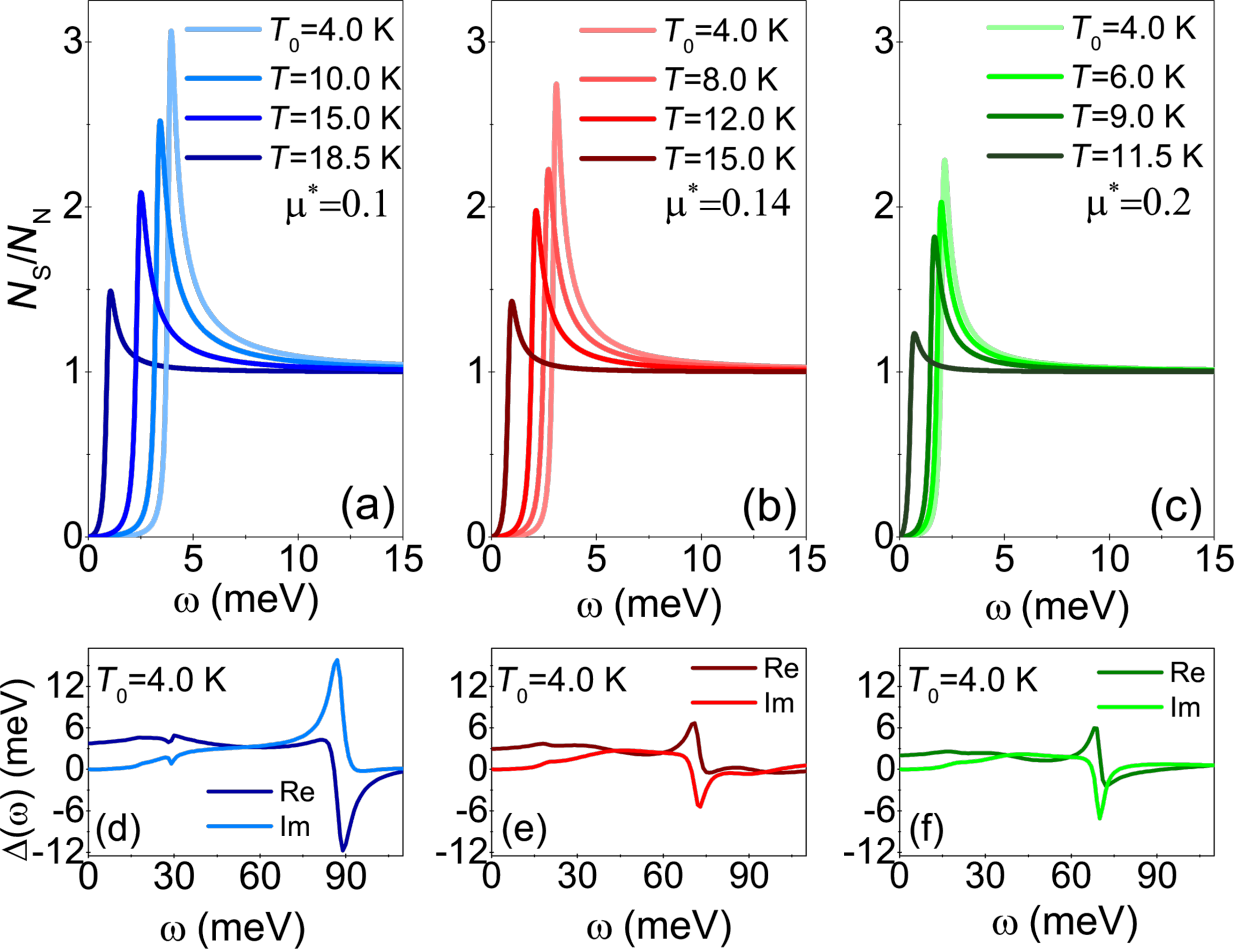
(a–c) Normalized density of states for the given temperatures. (d–f) The form of the order parameter on the real axis calculated for *T* = 4 K. The results were obtained in the framework of the LOVC model.

Knowing the full dependence of the order parameter on the Matsubara frequency, we determined the normalized density of states:

[7]
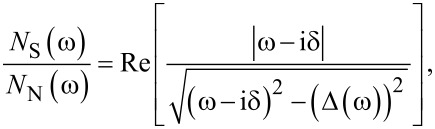


where the pair breaking parameter δ equals 0.15 meV. We calculated the value of Δ(ω) by continuing the function Δ*_n_* on the real axis [82]. The results obtained by using the LOVC approach for *N*_S_(ω)/*N*_N_(ω) are given in Figure 3a–c. The presented curves can also be determined on the basis of the data obtained using the tunneling junction. Hence, any experimental results directly relate to the predictions of the Eliashberg formalism taking into account the effect of vertex corrections. Additionally, in Figure 3d–f we plotted the form of the order parameter on the real axis (*T* = 4 K). The real part of the function Δ(ω) specifies the physical value of the order parameter, which can be calculated using the equation Δ(*T*) = Re[Δ(ω = Δ(*T*))] [62]. In the present case, we obtained values that differ from Δ*_n=_*_1_ by no more than 10^−2^%. This result proves that the analytical continuation was correct. The imaginary part of Δ(ω) determines the damping effects. One can see that at low frequencies, where Im[Δ(ω)] = 0, these effects do not occur. From the physical point of view, this means the infinite lifetime of the Cooper pairs. Above the frequency ω ≈ 15 meV, both the real and imaginary part of the order parameter function have a complicated course. This fact results directly from the complicated shape of the Eliashberg function, which models the electron–phonon interaction in the Li-hBN system.

Let us now discuss the effect of vertex corrections on the electron band mass (*m_e_*). To do this, it is necessary to use the formula 
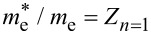
, where 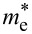 is the effective electron mass.

**Figure 4:**
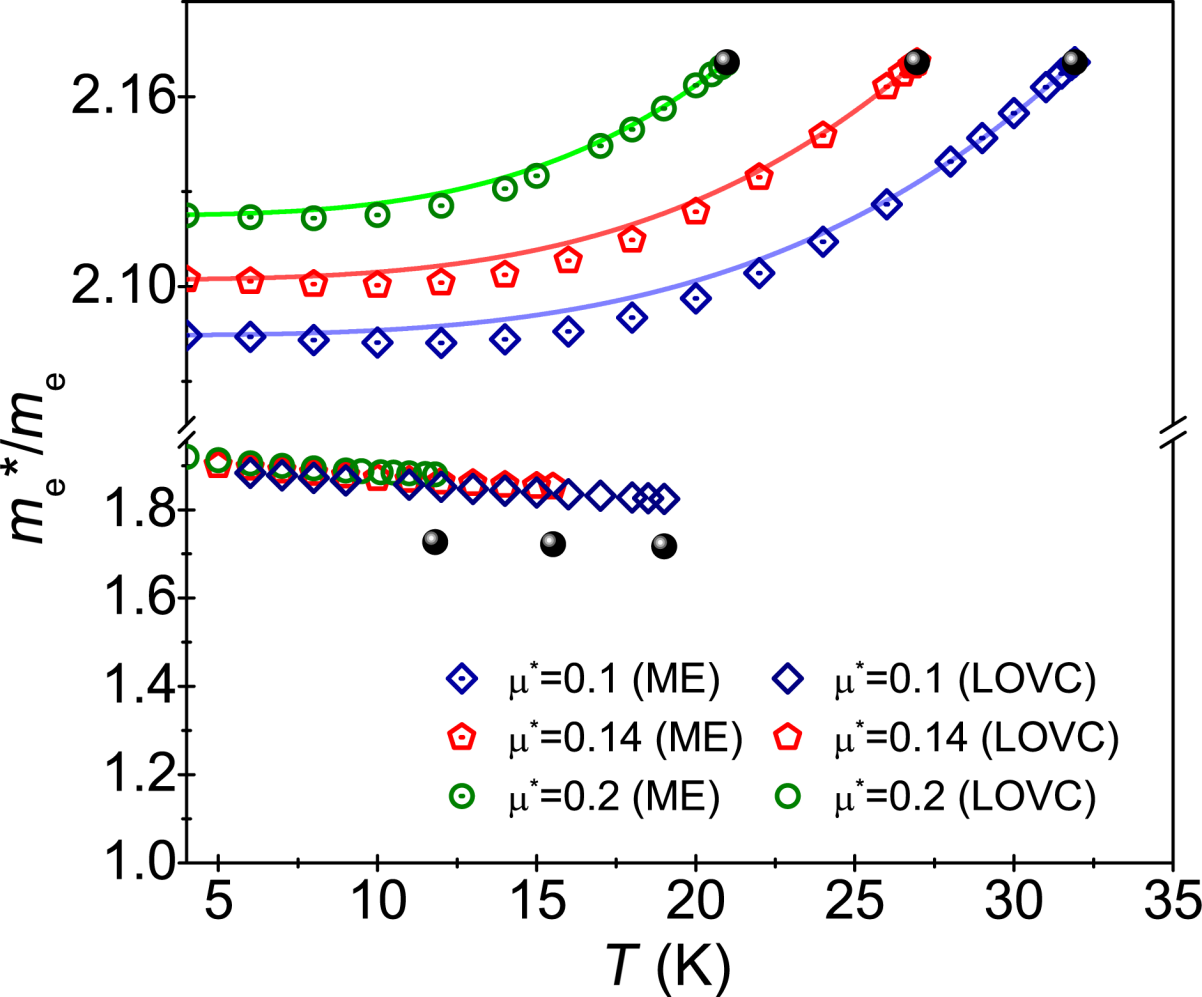
The ratio of the electron effective mass to the electron band mass as a function of temperature. The results were obtained in the framework of LOVC model, ME model, and Equation 9. The lines for ME results can be reproduced using the formula: 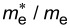 = [*Z_n=_*_1_(*T*_C_) − *Z_n=_*_1_(0)](*T*/*T*_C_)^Γ^ + *Z_n=_*_1_(0), where *Z_n=_*_1_(0) = *Z_n=_*_1_(*T*_0_).

The results obtained on the basis of the Eliashberg equations are presented in Figure 4. It is easy to see that the effective mass of the electron is almost twice as high as the electron band mass, with 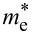 depending very slightly on the temperature. The vertex corrections lower the value of 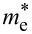 compared to the value predicted under ME formalism. If the temperature equals the critical temperature, this effect can be characterized analytically. Based on Equation 2, we obtained

[8]
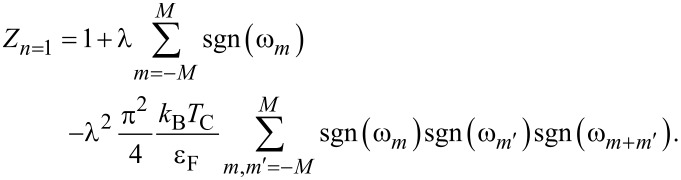


Hence,

[9]
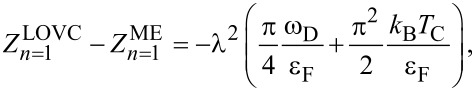


where 
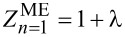
. We see that the lowest-order vertex corrections lower the effective mass the a greater extent when λ and ω_D_ are higher. It should be noted that in this case the critical temperature also increases. The values of 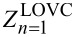 and 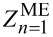, calculated on the basis of Equation 9, have been marked on Figure 4 using black spheres. We obtained a good agreement between numerical and analytical results.

## Discussion

A characterization of the superconducting state in Li-hBN was carried out with the help of the Eliashberg formalism taking into account the lowest-order vertex corrections. Due to the very high value of the ratio λω_D_/ε_F_, the question regarding the significance of higher-order vertex corrections arises. It turns out that no general answer can be given, as this would require the self-consistent solution of the Eliashberg equations taking into account the vertex corrections of all orders. However, one can give arguments that support the results presented in this paper:

(1) First of all, it should be noted that the very high value of the ratio λω_D_/ε_F_ does not mean that higher-order vertex corrections are equally or even more important than the lowest-order corrections. This is because the renormalization of the bare vertex amplitude *g* has the form [66]:

[10]
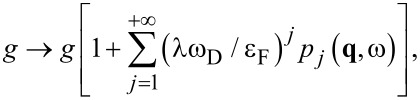


where the functions *p_j_*(**q**,ω) characterize the dependence of the vertex corrections on the momentum (**q**) and the frequency (ω) of the outgoing phonon. This means that not only the value of the ratio λω_D_/ε_F_ is important, but also the values of all functions *p_j_*(**q**,ω).

(2) The significance of the presented results can be thoroughly understood by analyzing the normal state within the LOVC model. In this case, the self-energy Σ(iω*_n_*) depends only on the wave function renormalization factor, because for a temperature equal to or higher than the critical temperature, the order parameter disappears. The values of the wave function renormalization factor obtained by us are easiest to analyze taking into account Equation 9, which should be written as

[11]
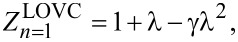


where


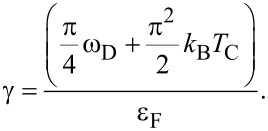


For the assumed values of the Coulomb pseudopotential, the parameter γ is in the range from 0.323 to 0.331. Therefore, for Li-hBN it is not high enough to lower 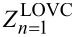 below 1, which would indicate the loss of stability of the examined compound [83].

Regarding literature results based on models that include the lowest-order vertex corrections [67,84–86], the sign of determined parameter γ is correct. This means that the static contribution of the vertex corrections to the normal self-energy has been qualitatively calculated in an appropriate manner. Due to the fact that Equation 9 reproduces well the numerical results (see Figure 4), the same can also be said about the self-consistent results. However, the values of the parameter γ for the models analyzed in [85] may differ significantly, which is associated with the assumed approximations. Our result is comparable with the estimates based on [67,85], where the γ parameter is equal to 0.317. However, it is lower than the values of 0.547, 0.685 and 1.07 that are obtained on the basis of [84–86]. On the other hand, the parameter γ determined by us exceeds the value of γ_W_ = 0.034 [87], which was obtained based on the Ward identity [85,88–89]. Note that Ward-type identities result from the conservation of the total charge and the total spin of fermions, as a result of which one can obtain the exact relationships between the self-energy and the vertex corrections. Nevertheless, the Ward identity considered in the context of superconducting states is an equation for two functions (scalar and vector) and, thus, allows for multiple solutions. Therefore, based on the results presented in [85,90–92] one can obtain the opposite value γ = −0.9.

To sum up, the presented LOVC model predicts a small decrease in the value of the wave function renormalization factor relative to other approaches based on the lowest-order vertex corrections. This result reduces the risk of stability loss of the examined system as a result of taking into account higher-order corrections. The estimated values of the parameter γ are very close to the value based on [67,85], and are higher than γ_W_, with the reservations made regarding the method based on the Ward identity.

(3) It is very difficult to justify our results for the values of temperatures lower than *T*_C_, where self-consistent calculations are required for φ*_n_* and *Z_n_*. Hence, we discuss the impact on our predictions of vertex corrections of the order of *g*^6^ (which corresponds to λ^3^). Due to the very complicated form of the considered contributions, we take into account the case |*T*_C_ − *T*| ≪ *T*_C_. This approach allows us to linearize the Eliashberg equations [62], which greatly simplifies the numerical analysis [77]. The contributions of the order of *g*^6^ to the Eliashberg equations were determined by using the Green thermodynamic formalism [93]. Note that the Migdal–Eliashberg approximation is based on the replacement of the mixed Green’s function


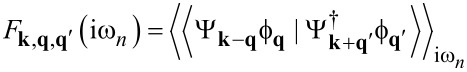


by the product of the full electron Green’s function *G***_k_**(iω*_n_*)(iω*_n_*) and the phonon propagator for the non-interacting phonons:


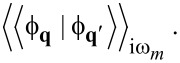


We have extended this step. In particular, we have strictly determined the equation of motion for *F***_k_**_,_**_q_**_,_**_q_**_'_(iω*_n_*)(iω*_n_*), and then repeated the procedure for all new unknown functions. Finally, based on the Wick theorem [94], we closed the obtained system of equations. The contribution of the order of *g*^6^ to the first Eliashberg equation can be written as presented in Equation 12. In the case of the equation for the wave function renormalization factor we obtained the expression in Equation 13.

[12]
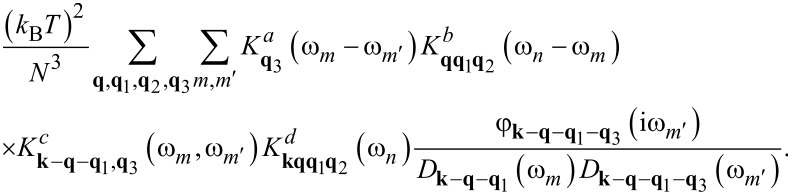


[13]
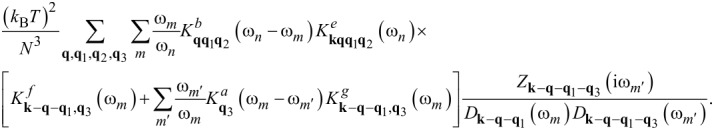


The explicit expressions for the kernels are given below:

[14]
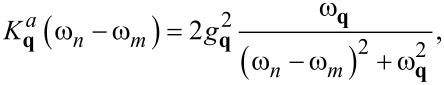


[15]
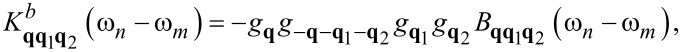


[16]
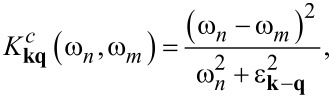


[17]
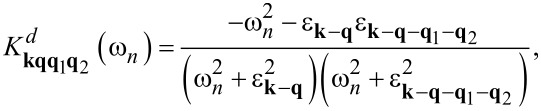


[18]
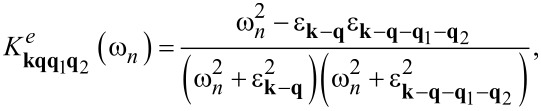


[19]
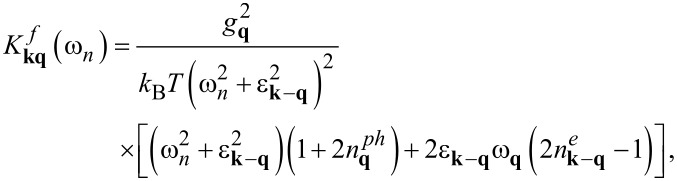


[20]
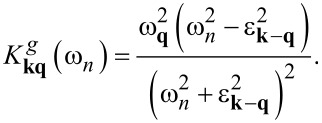


In Equation 14–Equation 20, g**_q_** means the electron–phonon matrix element, ω**_q_** represents the phonon energy, and ε**_k_** is the electron band energy. In addition, 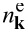 means the Fermi–Dirac function and 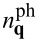 is the Bose–Einstein function. The symbol


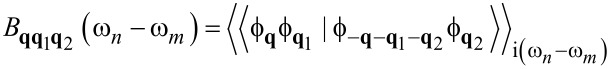


represents the higher-order phonon Green’s function. It was designated by us assuming no interaction between phonons. The function *D***_k_**(ω*_m_*) is given by the formula


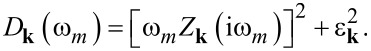


The isotropic form of contributions of the order λ^3^ (Equation 12 and Equation 13) was obtained by exchanging the wave vector summation by the energy integration with constant density of states, wherein some integrals can be calculated numerically. We do not give the explicit isotropic expressions, because they are very extensive. We performed numerical calculations for all considered values of the Coulomb pseudopotential. For μ* ∈ {0.1, 0.14, 0.2}, we obtained a reduction of the critical temperature 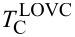, respectively, by 6.2%, 5.4% and 4.7%. This means that the vertex corrections of the order of λ^3^ do not significantly change the critical temperature values determined with the lowest-order vertex corrections. In our opinion, the analysis presented above clearly suggests that the critical temperature in Li-hBN is lower than 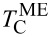.

**Figure 5:**
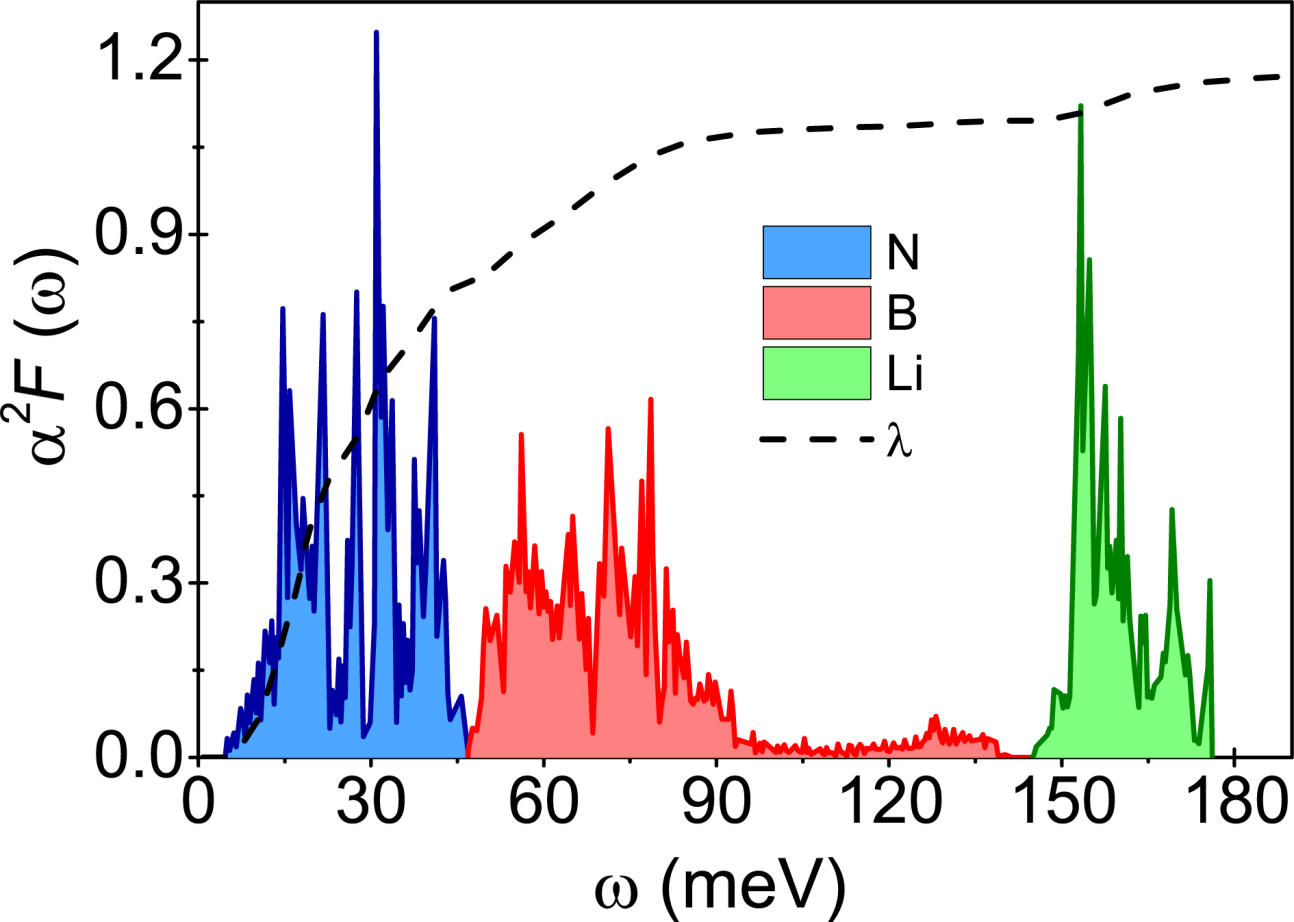
Eliashberg function α^2^*F*(ω) and electron–phonon coupling function 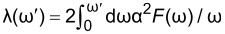 for Li-hBN. The results were obtained in [41]. The figure also indicates the contributions from nitrogen, boron and lithium, λ^N^ = 0.82, λ^B^ = 0.25, and λ^Li^ = 0.1, where λ^N^ + λ^B^ + λ^Li^ = 1.17.

In the last paragraph of this section, we discuss a possible way to increase the value of the critical temperature in Li-hBN. We believe that this is possible. To do this, consider the form of the Eliashberg function of Li-hBN (Figure 5). It can easily be seen that the Eliashberg function consists of two clearly separated parts (similar to the functions of hydrogen compounds [95–96]). In the low-frequency range (ω ∈ {4.59, 93.29} meV) nitrogen and boron contributions are important. In the frequency range from 145.16 to 176.13 meV, the electron–phonon interaction associated with lithium atoms dominates. These frequency ranges are separable, with the Eliashberg function taking very small values in the range from 93.29 to 145.16 meV. The above facts suggest that the composition of Li-hBN could be changed to significantly increase the values of the Eliashberg function in the range from 93.29 to 145.16 meV. Most likely by appropriate doping of the starting compound. However, this is not a simple task and requires DFT calculations.

Also striking is the possibility of substitution (at least partially) of lithium by hydrogen or of boron and nitrogen by heavier elements. In the first case, the increase in critical temperature could be associated with an increase of the Debye frequency (*T*_C_ ∼ ω_D_ and 
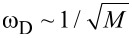
, while the hydrogen nucleus has a lower mass than the lithium nucleus). In the second case, the increase in *T*_C_ could result from the increase in the electron–phonon coupling constant (*T*_C_ ∼ exp(−1/λ). Contributions from heavier elements in the Eliashberg function are in the low-frequency range and are potentially more significant for λ. To find out, note that the electron–phonon coupling constant is defined by 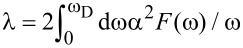.

## Conclusion

The superconducting state in Li-hBN is induced by electron–phonon interaction, which is characterized by the uncommonly high value of the ratio λω_D_/ε_F_ = 0.46. This means that the thermodynamic properties of the superconducting phase should be determined using a formalism explicitly including vertex corrections. Note that the very high value of the ratio λω_D_/ε_F_ is related to the quasi-two-dimensionality of considered system [41].

We showed that nonadiabatic effects significantly lower the critical temperature (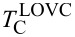 ∈ {19.1, 15.5, 11.8} K), compared to the results obtained in the framework of the Migdal–Eliashberg theory, 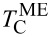 ∈ {31.9, 26.9, 21} K, for μ* ∈ {0.1, 0.14, 0.2}, respectively. In our opinion, there is no reason to believe that the critical temperature in Li-hBN exceeds 20 K, which certainly limits its applications. The vertex corrections of the order of λ^3^ slightly decrease 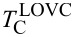.

Note that the low values of *T*_C_ occur in principle in the whole family of systems in which a honeycomb crystal structure plays an important role [5,47–49]. This structure, although fundamental for the properties of graphene, is unfavorable regarding superconducting states. The reason for this is that the van Hove singularity in the electronic density of states is considerably distant form the Fermi level [97]. This is not the case for a square lattice, where the van Hove singularity is very close or even at the Fermi level, which means that the value of *T*_C_ can increase by one order of magnitude [98].

Finally, let us note that from the point of view of fundamental research on phonon-induced superconducting states, the Li-hBN system seems to be very interesting because of the unusually high value of the ratio λω_D_/ε_F_, which is comparable to the value obtained for fullerene compounds [66,68]. Therefore, Li-hBN can be used to test the predictions of future theories that include vertex corrections in a fully self-consistent manner (both Matsubara frequencies and the electron wave vector **k**). We are currently investigating this issue extensively. Preliminary results for the ME formalism can be found in [99–100].
